# Advances in the Development of Nonpeptide Small Molecules Targeting Ghrelin Receptor

**DOI:** 10.1021/acs.jmedchem.1c02191

**Published:** 2022-02-14

**Authors:** Gianfabio Giorgioni, Fabio Del Bello, Wilma Quaglia, Luca Botticelli, Carlo Cifani, E. Micioni Di Bonaventura, M. V. Micioni Di Bonaventura, Alessandro Piergentili

**Affiliations:** † School of Pharmacy, Medicinal Chemistry Unit, University of Camerino, Via Madonna delle Carceri, 62032 Camerino, Italy; § School of Pharmacy, Pharmacology Unit, University of Camerino, Via Madonna delle Carceri 9, 62032 Camerino, Italy

## Abstract

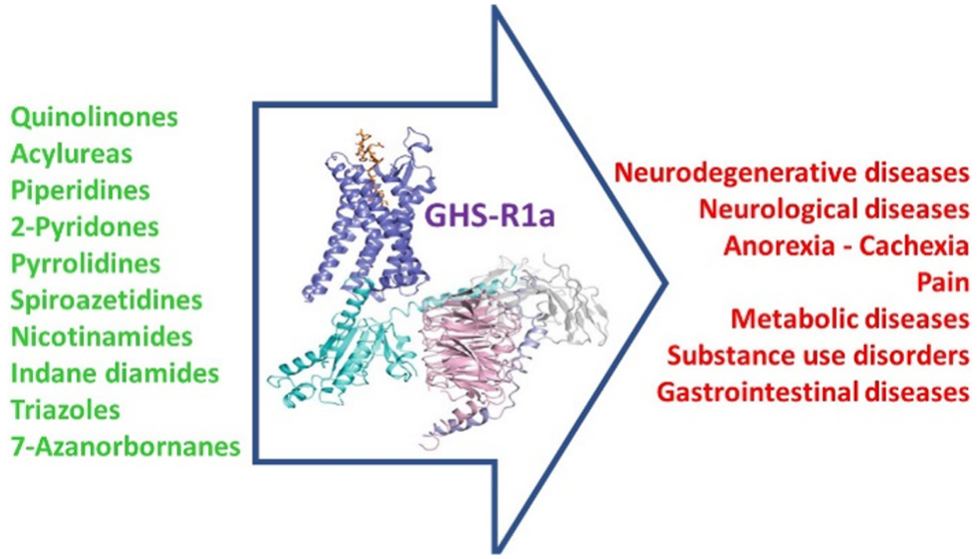

Ghrelin is an octanoylated peptide acting by the activation of the growth hormone secretagogue receptor, namely, GHS-R1a. The involvement of ghrelin in several physiological processes, including stimulation of food intake, gastric emptying, body energy balance, glucose homeostasis, reduction of insulin secretion, and lipogenesis validates the considerable interest in GHS-R1a as a promising target for the treatment of numerous disorders. Over the years, several GHS-R1a ligands have been identified and some of them have been extensively studied in clinical trials. The recently resolved structures of GHS-R1a bound to ghrelin or potent ligands have provided useful information for the design of new GHS-R1a drugs. This perspective is focused on the development of recent nonpeptide small molecules acting as GHS-R1a agonists, antagonists, and inverse agonists, bearing classical or new molecular scaffolds, as well as on radiolabeled GHS-R1a ligands developed for imaging. Moreover, the pharmacological effects of the most studied ligands have been discussed.

## 1 Introduction

**Figure 1:**
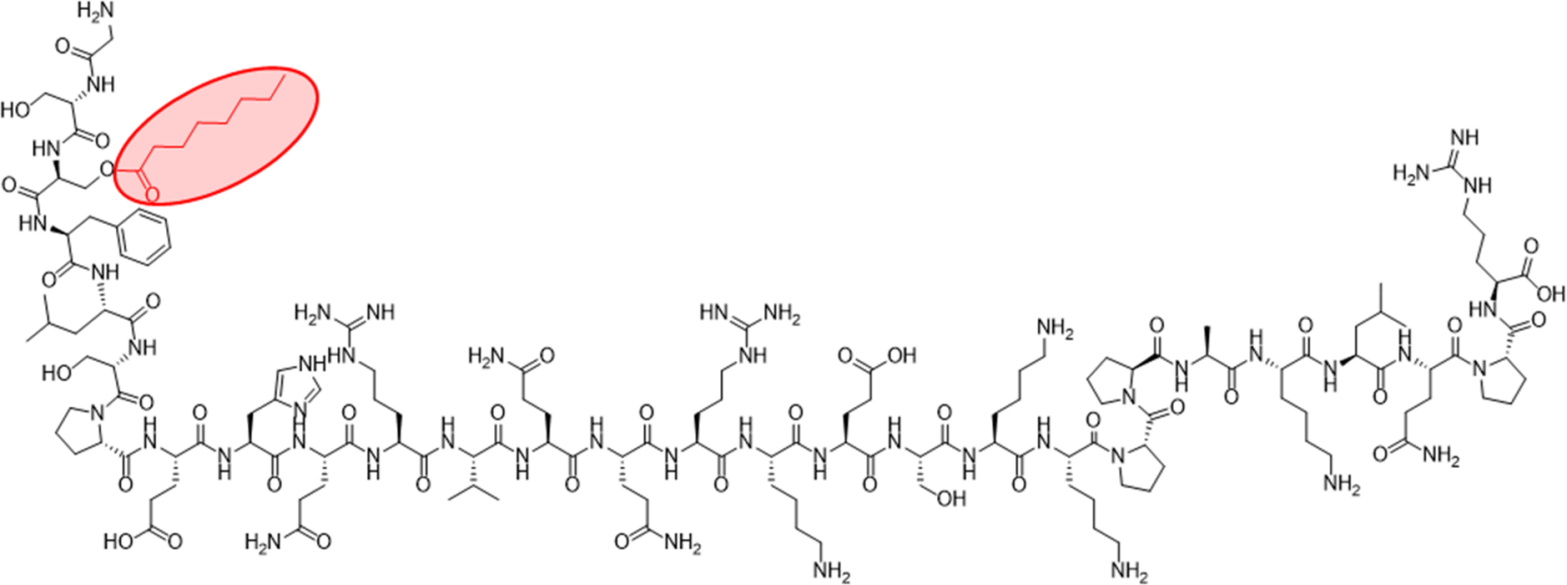
Structure of the octanoylated AG. The octanoyl group linked to Ser3 is colored in red.

Ghrelin, originally discovered in 1999, is a member of the group of growth hormone secretagogues (GHSs), well-known as hunger-stimulating hormone in humans. In plasma and in tissues, it is present in two main forms: the inactive 28 amino acid peptide desacyl-ghrelin (DAG) and the active acyl-ghrelin (AG, Figure 1), obtained through octanoylation at the Ser3 amino acid of DAG catalyzed by the enzyme ghrelin *O*-acyltransferase (GOAT).^1,2^ Ghrelin is mainly produced by the oxyntic glands in the stomach and is delivered in the bloodstream to reach the anterior pituitary gland, where it dose-dependently induces the release of the growth hormone (GH).^3,4^

Although a minority of circulating ghrelin undergoes octanoylation,^5^ only the octanoylated AG is able to activate the growth hormone secretagogue receptor, a G protein-coupled receptor (GPCR) known as GHS-R1a consisting of 366 amino acid residues.^6^ This receptor couples to a Gαq/11 protein, promoting Ca^2+^ mobilization from intracellular stores, through activation of the phospholipase C. It also signals through other G protein isoforms, including Gαi/o and Gα13 as well as β-arrestin scaffold proteins.^7−9^ Additional complexity in GHS-R1a signaling derives from the fact that this receptor shows one of the highest constitutive signaling activities in the GPCR family, evoking signals at around 50% of the maximal ghrelin response.^10,11^ Moreover, GHS-R1a can form homodimers and heterodimers with a variety of GPCRs, including GHS-R1b, an inactive splicing variant of GHS-R1a, serotonin 5-HT2c receptor, dopamine D1 and D2 receptors, somatostatin SST5 receptor, orexin OX1 receptor, melanocortin MC3 receptor, and cannabinoid CB2 receptor.^10,12−14^

Very recent studies have provided useful information about the structure of GHS-R1a bound to ghrelin,^15−18^ synthetic agonists,^16,18^ a neutral antagonist,^19^ or an inverse agonist,^20^ which will help the design of new GHS-R1a selective drugs.

This receptor is highly expressed in the central nervous system (CNS), mainly in the hypothalamus and pituitary gland, but also in the rafe nuclei, hippocampus, ventral tegmental area, and substantia nigra pars compacta.^12,21−23^ It is also localized in periphery and in particular in the spleen, pancreas, adrenal glands, and kidney.^12,24^ Moreover, GHS-R1a expression has been found in the cardiovascular system.^25^

The inactive splicing variant GHS-R1b is a five transmembrane domain protein composed of 289 amino acids that is not activated by ghrelin and lacks the ability to mobilize Ca^2+^.^26,27^

Since ghrelin activates only GHS-R1a, such a receptor represents an important target mediating several physiological functions. Indeed, AG fulfills roles, such as regulation of appetite level, stimulation of food intake, gastric emptying, body energy balance, glucose homeostasis, reduction of insulin secretion, and lipogenesis.^28,29^ On the contrary, DAG induces opposite effects interacting with an uncertain receptor.^30^ Together with the hypothalamic activities, the role of ghrelin system and the enzyme GOAT in food intake regulation is also related to the interaction with other neurotransmitter systems implicated in feeding management as well as to the expression of ghrelin receptors in extrahypothalamic sites.^31^ Ghrelin has also been reported to play a role in some neurological functions such as memory, fear, anxiety, depression, addiction, and alcohol intake.^32−36^ Moreover, AG stimulates GHS-R1a in the brain and induces anticonvulsant and neuroprotective effects, suggesting that it is a potential target for the treatment of neurodegenerative disorders, such as Parkinson’s and Alzheimer’s diseases.^26,37,38^ Ghrelin has also been discovered in heart cells, supporting the hypothesis of its cardiovascular effects and cardioprotective activity.^39^ It has recently been demonstrated that ghrelin can act directly on hepatocytes to stimulate lipogenesis and may serve as a marker and therapeutic target for nonalcoholic steatohepatitis.^40^

**Figure 2:**
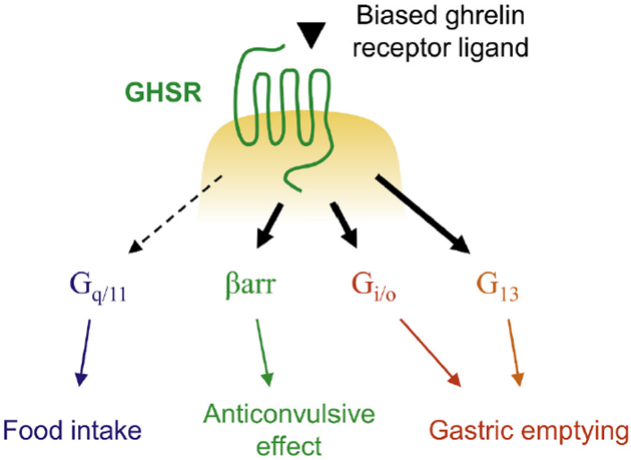
Different physiological effects mediated by distinct signaling pathways of GHS-R1a. Reproduced from ref (7) with permission from Elsevier.

Interestingly, recent studies report that different physiological responses of AG are evoked by distinct signaling pathways of GHS-R1a.^7,8,41,42^ Therefore, biased ligands endowed with functional selectivity might represent a promising therapeutic strategy for the treatment of diseases dependent on the modulation of a specific signaling pathway, avoiding potential side effects associated with the modulation of other pathways. For instance, functionally selective ligands able to activate β-arrestin pathway might be potentially useful as antiepileptic agents, while the selective activation of Gi/o and G13 might be beneficial for gastric empying (Figure 2).

The melanocortin receptor accessory protein 2 (MRAP2) has been identified as an important modulator of the ghrelin-GHS-R1a system, able to potentiate AG-stimulated signaling both *in vitro* and *in vivo*. In particular, MRAP2 evoked biased signaling downstream of AG-mediated GHS-R1a activation by potentiating Gαq/11-dependent signaling and inhibiting β-arrestin recruitment. Moreover, MRAP2 suppressed the high ligand-independent activity of GHS-R1a.^43,44^

Liver-expressed antimicrobial peptide 2 (LEAP2), a 40-residue cationic peptide predominantly localized in the small intestine and liver, has recently been described as an endogenous GHS-R1a antagonist.^45^ Both LEAP2 and its N-terminal portion behave as GHS-R1a inverse agonists and competitively antagonize ghrelin-induced Ca^2+^ mobilization and inositol-1-phosphate (IP) production. They have also been demonstrated to inhibit AG-induced food intake in mice.^46^

The considerable attention of researchers on the ghrelin system is demonstrated by the large number of paper published in the past decade and a half. Running a search in Scopus (www.scopus.com) for the term “ghrelin” in article titles and limiting the results to the articles published only in 2020 and 2021, 478 document results have been found, including 43 review articles.

The broad spectrum of processes involving ghrelin-dependent pathways opens the opportunity to evaluate new potentially therapeutic approaches for the treatment of several disorders.^10,31,38,47−49^ Thus, agonists, antagonists, and inverse agonists of the GHS-R1a have been developed over the years.^50−53^ Moreover, ghrelin signaling can be inhibited by blocking GOAT activity. Even if this way has not been fully explored yet, it seems to be another promising drug target, as exhaustively described in very recent review articles.^54,55^

Regarding the receptor ligands, nonpeptide compounds are particularly interesting, due to the very low stability of peptide-based structures, including the endogenous ligand ghrelin, that are subjected to high gastrointestinal degradation.^56^ Therefore, though several peptide derivatives have been reported as potent GHS-R1a ligands,^46,57,58^ this perspective is focused on the development of recent small molecules acting as GHS-R1a agonists, antagonists, and inverse agonists and bearing classical or new molecular scaffolds. G-protein and β-arrestin signaling bias will be considered. Moreover, GHS-R1a ligands developed for positron emission tomography (PET) imaging will be reported. Finally, the pharmacological effects of the most studied ligands will be discussed.

## 2 Structure of GHS-R1a

Solution-state nuclear magnetic resonance (NMR) combined with advanced molecular modeling have provided useful information about the conformation of GHS-R1a bound to ghrelin in its active and inactive state. In particular, the octanoyl chain of AG seems to be required to form a well-defined hydrophobic core and to favor access of AG to the binding pocket. The results have also demonstrated some degree of both conformational and positional local dynamics of AG even after it reaches its binding pocket.^15^ Solid-state NMR in combination with site-directed mutagenesis and modeling studies have also been performed to investigate the structural basis of GHS-R1a bound to ghrelin. The results have revealed an extended binding surface for this interaction and support the evidence that AG binds the receptor through two sites.^17^

**Figure 3:**
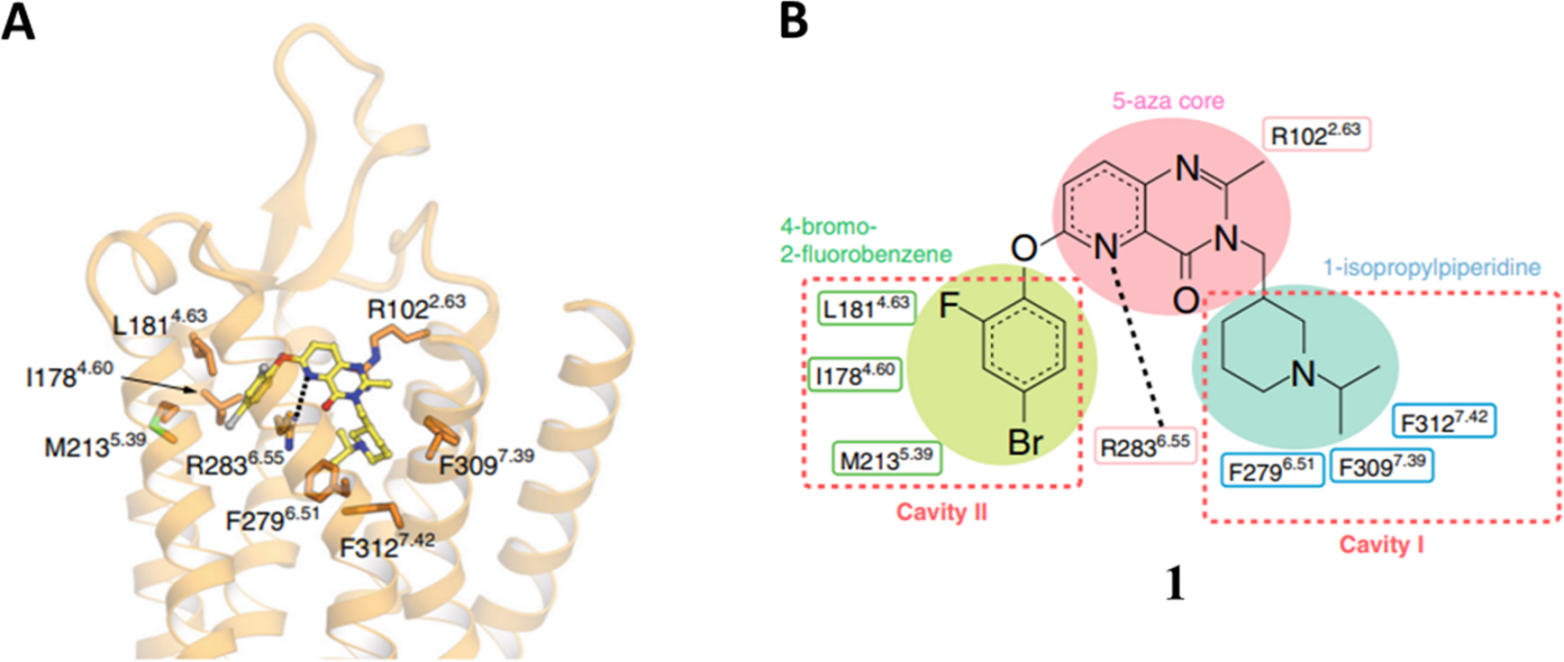
Binding mode of compound **1**. (A) Side chain interactions within 4.0 Å residues are shown in stick representation. Hydrogen bonds are shown as black dashed lines. (B) Schematic representation of the interactions between GHS-R1a and compound **1**, analyzed using Discovery Studio 2016. The black dot line indicates a hydrogen bond. Reproduced from ref (19), which was published under a Creative Commons Attribution 4.0 International (CC BY 4.0) License.

Recently, the crystal structure of GHS-R1a in complex with the antagonist **1** has also been determined (Figure 3).^19^ The results have revealed that the binding pocket is characterized by a wide gap between TM6 and TM7 and is bifurcated into two cavities by a salt bridge between Glu124 and Arg283 (Figure 3B). The larger cavity has been named cavity I, and the smaller one cavity II. Mutagenesis studies have suggested that the cavity I is more important for the binding of AG.

**Figure 4:**
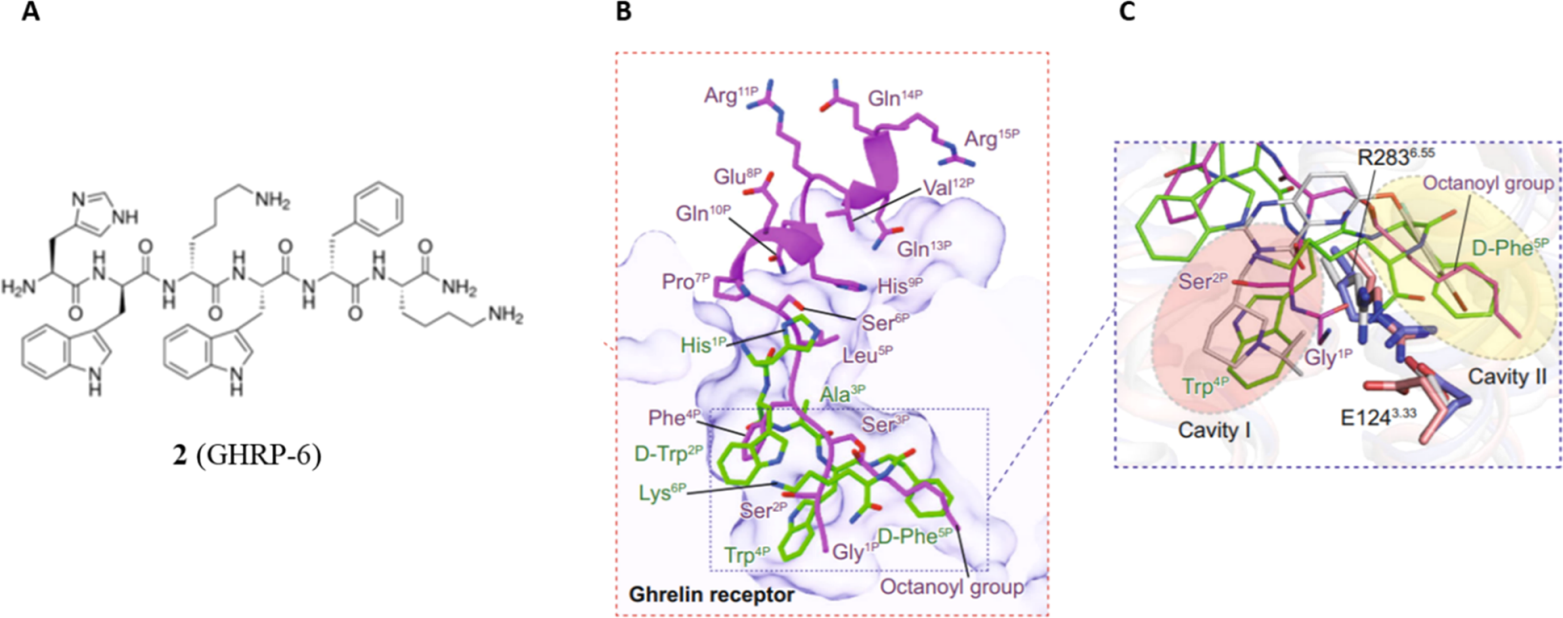
(A) Chemical structure of compound **2** (GHRP-6). (B) Binding poses of ghrelin and **2**. (C) The binding pocket of GHS-R1a is bifurcated into two cavities by a salt bridge between Glu124 and Arg283. Ghrelin is shown in magenta, ghrelin-bound GHS-R1a in slate blue, compound **2** in green, and **2**-bound GHS-R1a in salmon. **1**-bound GHS-R1a (PDB 6KO5) is colored in gray. Adapted from ref (16), which was published under a Creative Commons Attribution 4.0 International (CC BY 4.0) License.

In another study, the analysis of cryo-electron microscopy structures of ghrelin and the peptide agonist GHRP-6 (**2**) in complex with Gq-coupled GHS-R1a has revealed a unique binding pocket for the octanoyl group of AG, which favors its correct positioning to activate the receptor (Figure 4).^16^

In this structure, the octanoyl group is located at cavity II but not at cavity I. This result is different from those reported in previous modeling studies.^15,17,19^

**Figure 5:**
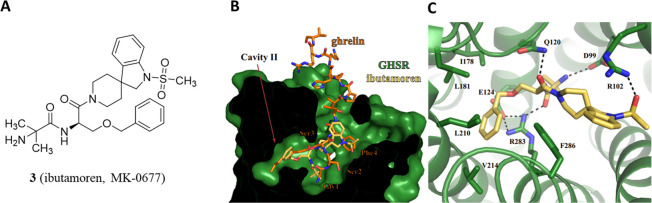
(A) Chemical structure of compound **3** (ibutamoren, MK-0677). (B) Alignment of ghrelin and ibutamoren. GHS-R1a bound to **3** is colored in green. Compound **3** is shown as yellow sticks. C) Compound **3** is in the binding pocket. Adapted from ref (18), which was published under a Creative Commons Attribution 4.0 International (CC BY 4.0) License.

Accordingly, the reported cryo-electron microscopy structures of Gi-coupled GHS-R1a in complex with ghrelin and the nonpeptide small molecule ibutamoren (MK-0677, **3**) (Figure 5) have shown that the peptide moiety of AG mainly occupies cavity I, while the octanoyl moiety is accommodated at cavity II, adopting an extended conformation.^18^ Compound **3** occupies both the cavities at the bottom area of the binding pocket, mimicking the first four residues of AG (including the octanoyl moiety).

**Figure 6:**
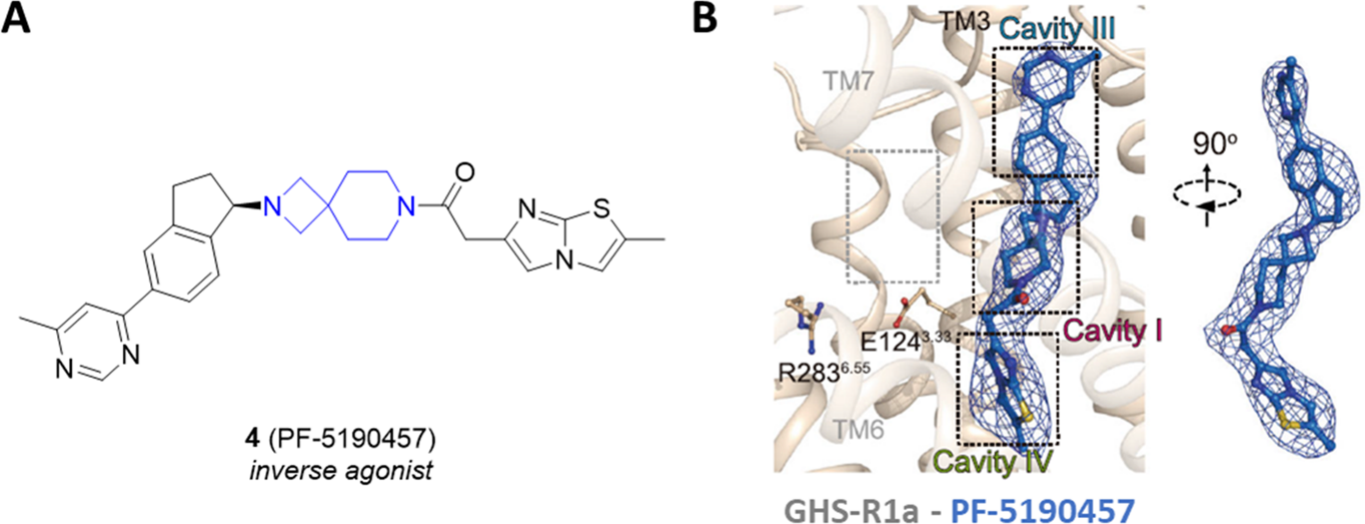
(A) Chemical structure of the GHS-R1a inverse agonist **4** (PF-5190457). (B) The detailed binding mode of **4** (marine blue sticks) in the orthosteric pocket of the GHS-R1a. Adapted from ref (20), which was published under a Creative Commons Attribution 4.0 International (CC BY 4.0) License.

Very recently, the crystal structure of GHS-R1a in complex with the inverse agonist PF-5190457 (**4**) together with a cryo-electron microscopy structure of the Go-coupled GHS-R1a in complex with AG highlighted that the inverse agonist **4** shows a binding mode different from those of both neutral antagonists and agonists (Figure 6).^20^ In particular, a hydrophobic cluster and a polar network seems to be required for the receptor activation and constitutive activity.

Altogether, these structural studies have discussed active and inactive states of GHS-R1a and have shed light on the different binding modes of agonists, neutral antagonists, and inverse agonists, improving the knowledge of the molecular mechanism for GHS-R1a recognition and activation and providing useful information for the structure-based design of new GHS-R1a selective drugs.

## 3 Medicinal Chemistry of GHS-R1a Ligands

### 3.1 GHS-R1a Agonists

**Figure 7:**
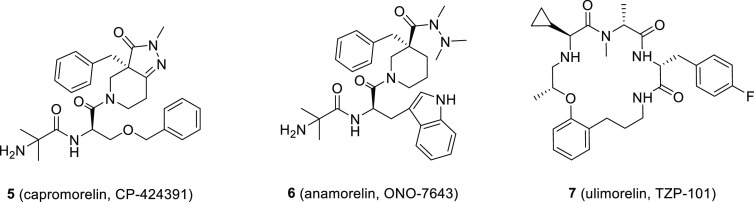
Chemical structure of the GHS-R1a agonists **5**–**7**.

Over the years, several GHS-R1a agonists have been reported and developed for the treatment of disorders related to the dysregulation of the functions mediated by GHS-R1a. Some of them, such as **3** (Figure 5), capromorelin (CP-424391, **5**), anamorelin (ONO-7643, **6**), and ulimorelin (TZP-101, **7**) (Figure 7), have reached advanced clinical trials for gastrointestinal diseases, cancer cachexia, and sarcopenia (see section 4).

**Figure 8:**
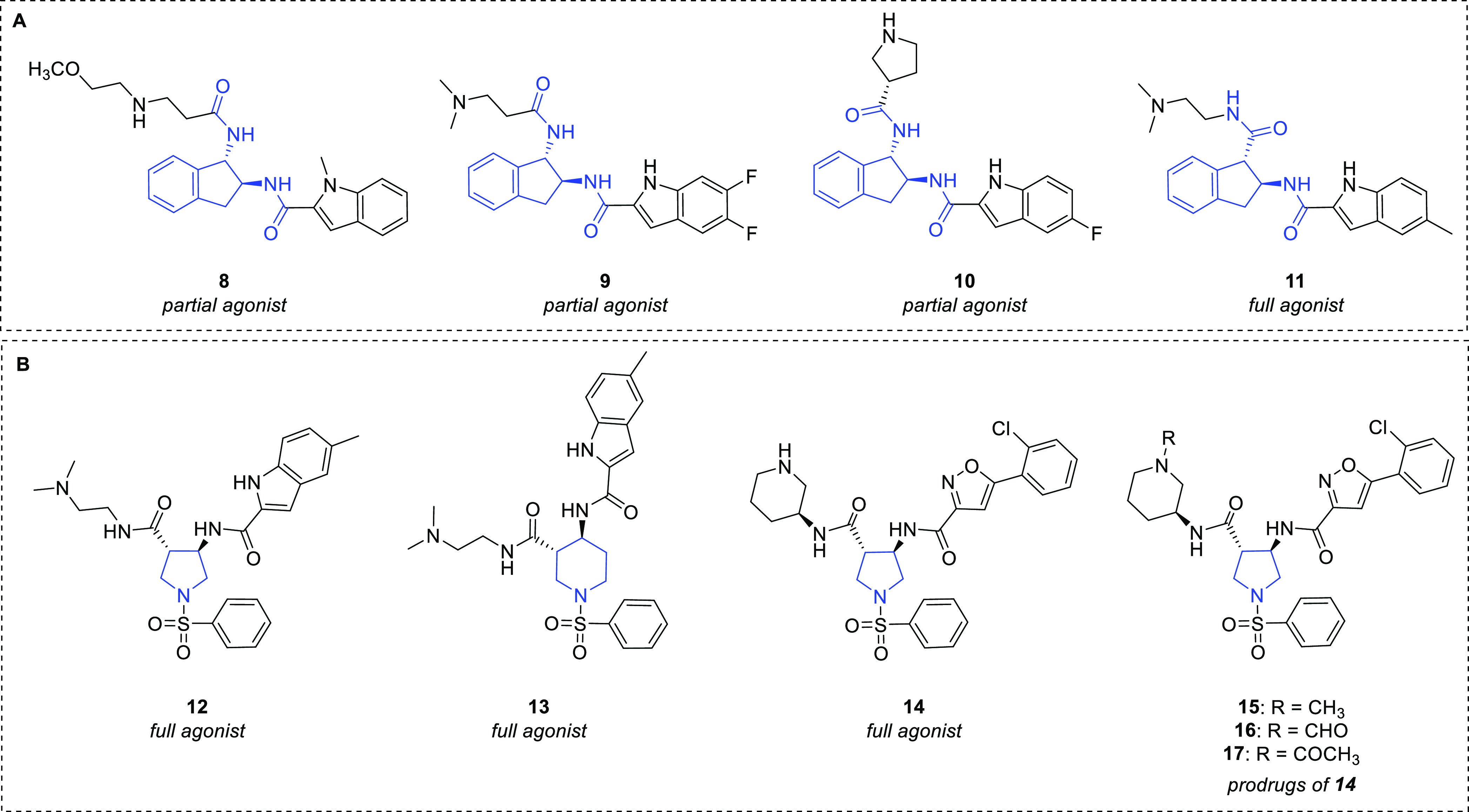
Chemical structure of (A) the indane diamide GHS-R1a agonists **8**–**11** and (B) the pyrrolidine and piperidine GHS-R1a agonists **12**–**17**.

Recently, new agonists with different molecular scaffolds are emerging as potential tools to treat a variety of clinical conditions. A high-throughput screening (HTS) approach on AstraZeneca’s library, followed by hit to lead generation, led to the discovery of a series of indane diamides behaving as GHS-R1a partial agonists (**8**–**10**) with submicromolar potency (Figure 8A).^59^ From a subsequent lead optimization strategy, an interesting modulation of the biological profile from partial to full agonism was obtained. In particular, an extensive SAR study led to the identification of the potent druglike GHS-R1a full agonist **11** (EC_50_ = 1.6 nM; *E*_max_ = 89%) (Figure 8A),^59^ which was devoid of significant hERG channel inhibition. This compound showed adequate pharmacokinetic (PK) profile, displaying long half-life and limited brain penetration and increased insulin-like growth factor-1 (IGF-1) secretion in dogs. This effect may be useful in cachexia, which is characterized by impairment of skeletal muscles and is associated with several chronic diseases such as chronic obstructive pulmonary disease, cancer, and acquired immunodeficiency syndrome. Unfortunately, compound **11** also showed off target activity toward the mu1-opioid receptor that stopped its further development.

Later, a new series of derivatives retaining the key pharmacophoric features of indanes and showing improved selectivity and PK profiles was designed and developed.^60^ In particular, the potent pyrrolidine and piperidine full agonists **12** (EC_50_ = 0.79 nM; *E*_max_ = 93%) and **13** (EC_50_ = 0.79 nM; *E*_max_ = 98%) (Figure 8B), respectively, structurally related to **11**, have been reported. Their optimization led to the identification of the highly potent and selective compound **14** (EC_50_ = 0.40 nM; *E*_max_ = 98%) (Figure 8B), which showed sustained dose-dependent activity in a dog IGF-1 model, long and suitable PK, and safety profile.^60^ However, **14** was not considered a clinically suitable candidate as it was poorly absorbed when administered per os in rodent species owing to a combination of low permeability and P-glycoprotein (Pgp)-mediated efflux. In the effort to increase the permeability and reduce the affinity for Pgp, derivatives **15**–**17** (Figure 8B) were also prepared and studied.^60^ They can be considered potential prodrugs of **14**, which was identified as their major metabolite in human, dog, and rat hepatocytes. However, due to the too low detected levels of **14**, derivatives **15**–**17** were not progressed as prodrugs. Studies focusing on the modifications to the core structure are still in progress.

Some “privileged structural motifs”, including 2-pyridone, quinolone, and 7-azanorbornane, have also been used as scaffolds of compounds acting as potent GHS-R1a agonists.

**Figure 9:**
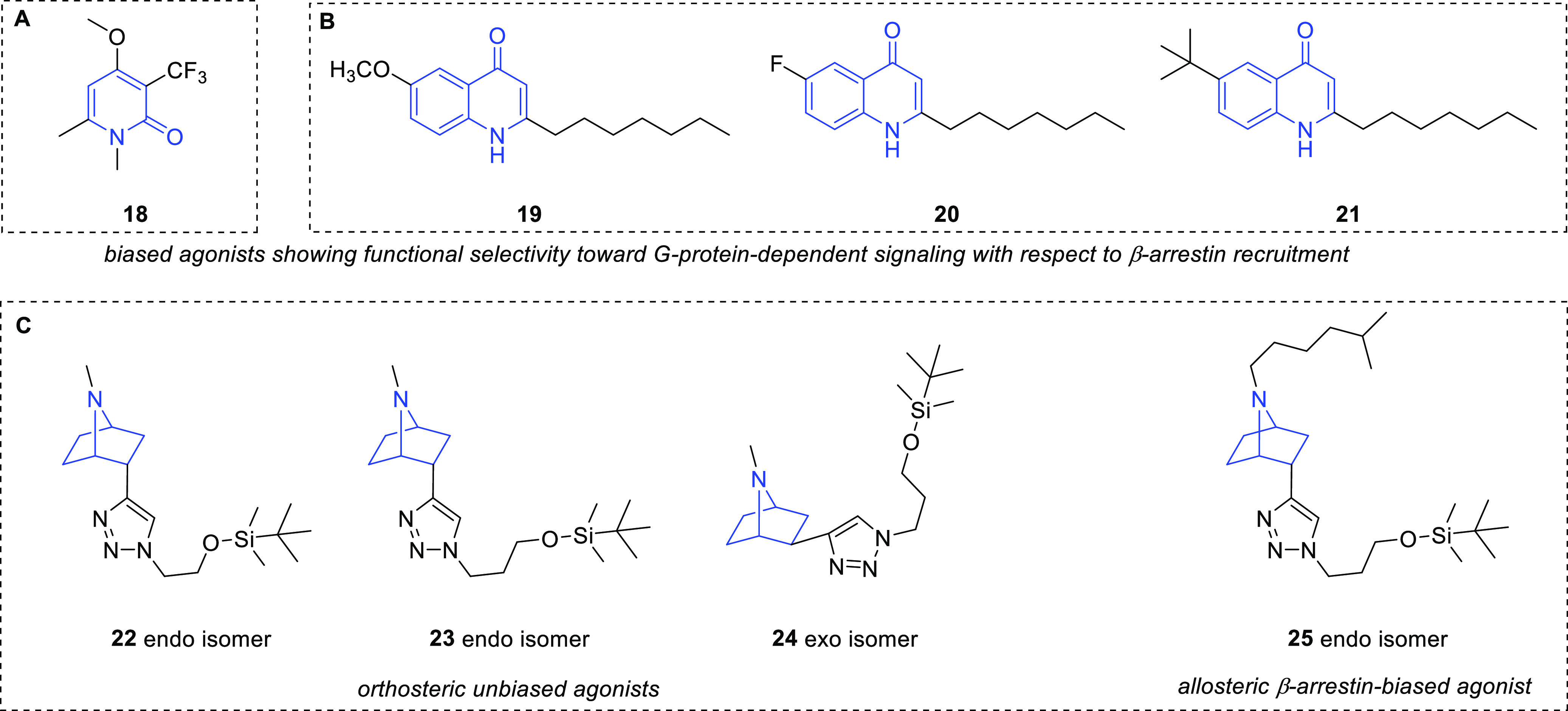
Chemical structure of (A) the 2-pyridone GHS-R1a agonist **18**, (B) the quinolone GHS-R1a agonists **19**–**21**, and (C) the 7-azanorbornane GHS-R1a agonists **22**–**25**.

2-Pyridones were selected for a screening program to identify nonpeptidic small molecules able to potently activate GHS-R1a *in vitro* in both transfected human cells and mouse hypothalamic cells and to induce *in vivo* orexigenic effects.^56^ In particular, the lead compound **18** (Figure 9A) showed a significantly increased food intake following intraperitoneal administration in male C57BL/6J mice and may represent a potential tool for the treatment of cachexia. Recently, this compound has been reported as a biased agonist that showed functional selectivity toward G-protein-dependent signaling, being able to increase Ca^2+^ influx, without affecting GHS-R1a internalization or increasing β-arrestin recruitment.^61^

Another bioversatile scaffold, that has been considered a core structure of potent GHS-R1a agonists, is the quinolinone nucleus. Such a privileged structure is present in synthetic compounds endowed with different pharmacological properties, including antimicrobial, antiallergenic, and anticancer activities. Sixteen quinolones, characterized by various substituents in positions 3, 6, 7, and 8 and alkyl chains of different lengths in position 2, were investigated for their potential to modulate GHS-R1a activity.^62^ Based on an intracellular calcium mobilization test in both transfected human cells and mouse hypothalamic cells, the hit compounds **19**–**21** (Figure 9B), characterized by a CH_3_O, F, or (CH_3_)_3_C substituent, respectively, in position 6 and an *n*-heptyl chain in position 2, emerged as the most promising agonists (EC_50_ = 4.5 μM, *E*_max_ = 121% for **19**; EC_50_ = 2.2 μM, *E*_max_ = 95% for **20**; EC_50_ = 73 μM, *E*_max_ = 102% for **21**) with an effect like that induced by ghrelin (EC_50_ = 0.3 μM, *E*_max_ = 100%). Moreover, they were not able to induce β-arrestin recruitment and subsequent GHS-R1a internalization and desensitization and, therefore, might be considered functionally selective GHS-R1a agonists. Further studies are needed to investigate the role of this functional selectivity in mediating the potential of the quinolone GHS-R1a ligands as orexigenic agents in cachexia and associated disorders.

A series of 22 compounds with “druglike” properties and bearing the sp^3^-rich 7-azanorbornane scaffold was prepared by click chemistry.^63^ Among them, the hit derivatives **22**–**24** (Figure 9C), bearing a *tert*-butyldimethylsilyloxyalkyl group on a triazole ring, dose-dependently activated GHS-R1a. This effect was contrasted by pretreatment with a competitive GHS-R1a antagonist, demonstrating that they bind to the orthosteric site of the receptor. Interestingly, further efforts devoted to the structure optimization concerning the substituent on the N7 of the azanorbornane scaffold of the most active compound **23** led to the discovery of the putative β-arrestin-biased superagonist **25** (Figure 9C).^64^ Since the effect of **25** was only partially blocked by a competitive antagonist, its binding to an allosteric site was also hypothesized. Moreover, this study suggests that, despite its easy-to-perform nature, the calcium assay alone might not be sufficient to completely highlight all the remarkable features of the GHS-R1a ligands.

### 3.2 GHS-R1a Antagonists and Inverse Agonists

The GHS-R1a antagonists and inverse agonists published and patented so far bear different molecular scaffolds. Many of them have been accurately described in previous review articles.^50,51^ The most recently discovered compounds will be discussed in this section.

**Figure 10:**
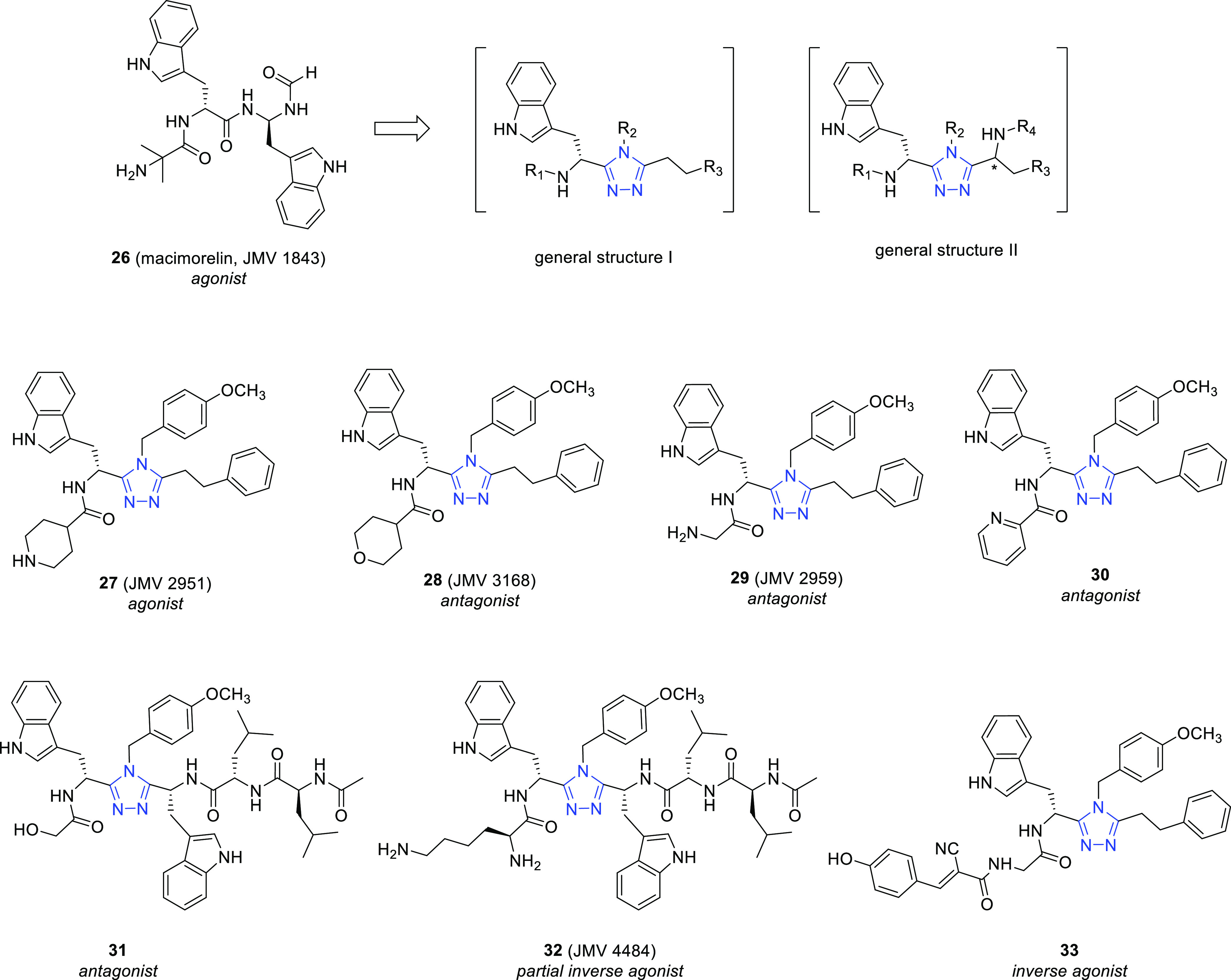
Chemical structure of the pseudopeptide GHS-R1a agonist **26** and the structurally related triazole ligands **27**–**33**, belonging to the general structures I and II.

Starting from the known pseudopeptide macimorelin (JMV 1843, **26**) (Figure 10), acting as a potent GHS-R1a agonist,^65^ a series of structurally related small molecules bearing the 1,2,4-triazole scaffold was developed. Interesting results emerged from structure–activity relationship (SAR) studies of these compounds. Many of them present an α-aminoisobutyryl moiety as an R_1_ substituent of the general structure I (Figure 10). However, the replacement of such a moiety with different groups led to GHS-R1a ligands endowed with high affinity and different functional behavior. The isonipecotyl compound **27** (JMV 2951) (Figure 10) proved to be an agonist (EC_50_ (Ca^2+^) = 1.6 nM). Interestingly, the replacement of the piperidine NH of **27** with an oxygen atom, yielding the isostere JMV 3168 (**28**, IC_50_ (Ca^2+^) = 60 nM). (Figure 10), modulated the profile from GHS-R1a agonism to antagonism.^66−68^

The glycyl and 2-picolinic derivatives **29** (JMV 2959) and **30** (Figure 10), respectively, also behaved as potent GHS-R1a antagonists (IC_50_ = 32 nM and 0.7 nM for **29** and **30**, respectively). From a PK point of view, **30** showed a better profile than **29**, displaying a slow clearance and a long drug exposure to the body. Starting from compound **30**, an extensive SAR study, performed by modifying the position of the pyridine ring and introducing substituents on it, indicated that the ortho position of the N atom is crucial for the affinity and various substituents (F, CH_3_, OCH_3_) are well tolerated.^69^

A subsequent study, performed on this 1,2,4-triazole series and concerning the introduction of a second chiral center, led to compounds of general structure II (Figure 10), endowed with nanomolar affinities for GHS-R1a.^70^ Interestingly, while most of the compounds were GHS-R1a agonists, compound **31** behaved as a neutral antagonist (*K*_i_ = 3 nM, *E*_max_ = 0%) and **32** (JMV4484) (Figure 10) as a partial inverse agonist (*K*_i_ = 3 nM, EC_50_ = 70 nM, *E*_max_ = −37%) with a potency similar to that of the hexapeptide KwFwLL-NH_2_ (*K*_i_ = 255 nM, EC_50_ = 100 nM, *E*_max_ = −55%) used as reference compound.^71^

Very recently, compound **29** has been used as a model for the preparation of a series of 45 new 3,4,5-trisubstituted 1,2,4-triazole ligands,^72^ among which 17 compounds behaved as GHS-R1a inverse agonists with a potency similar to that of the reference compound K-(D-1-Nal)-FwLL-NH_2_.^73^ Moreover, 4 inverse agonists showed an efficacy even higher than that of the first inverse agonist analog of substance P ([(D)Arg1,(D)Phe5,(D)Trp7,9,Leu11]-substance P), often referred in the literature as SPA (*E*_max_ = 78%).^74^ Derivative **33**, one of the most promising compounds (Figure 10), was selected for *in vitro* and *in vivo* studies, demonstrating to block the inhibitory action of ghrelin on insulin secretion in rat-isolated pancreatic islets and to reduce food intake induced by ghrelin in mice.^72^ Such a result confirms the suitability of the properly substituted 1,2,4-triazole scaffold for the development of inverse agonists potentially useful for the treatment of obesity-related metabolic diseases.

**Figure 11:**
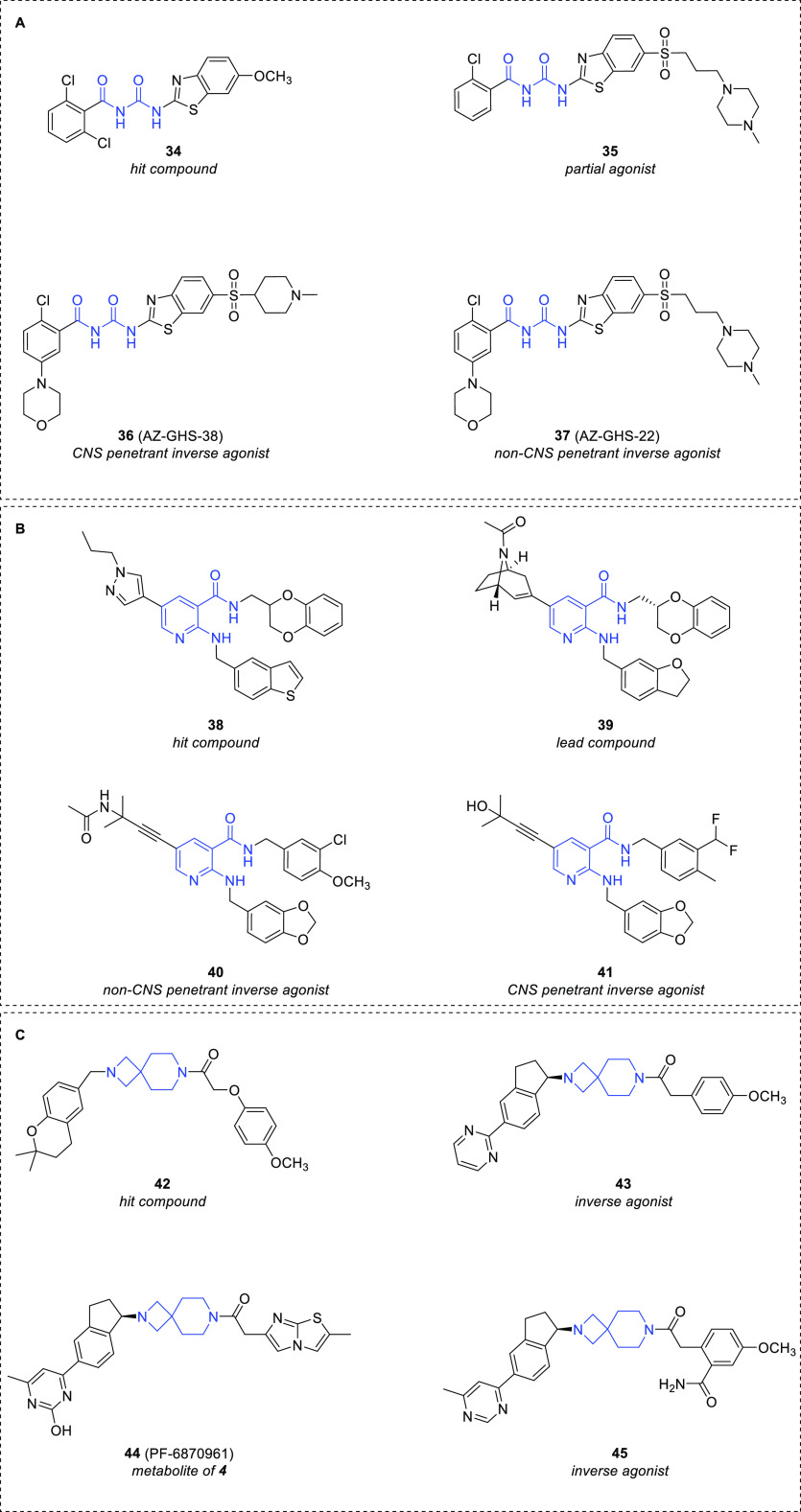
Chemical structure of (A) the acylurea GHS-R1a ligands **34**–**37**, (B) the 2-aminoalkyl nicotinamide GHS-R1a ligands **38**–**41**, and (C) the spiro-azetidine-piperidine GHS-R1a ligands **42**–**45**.

Inverse agonists bearing other molecular scaffolds, including acylurea, spiro-azetidine-piperidine, and nicotinamide, have been identified by an HTS approach, followed by chemical optimization through SAR studies. A HTS campaign on the AstraZeneca compound library led to the acylurea hit **34** (Figure 11A), which showed moderate affinity for GHS-R1a (IC_50_ (affinity) = 210 nM).^75^ The removal of one chlorine atom and the substitution of the 6-methoxy group with a (3-(4-methylpiperazin-1-yl)propyl)sulfonyl side chain afforded the partial agonist **35** (IC_50_ (affinity) = 1.3 nM) (Figure 11A) which showed higher affinity than **34**.

Further structural optimization led to the modulation of the biological profile from partial to inverse agonism and to the optimization of physicochemical and PK properties. In particular, the CNS penetrant inverse agonist **36** (AZ-GHS-38) (IC_50_ (affinity) = 0.77 nM) and the non-CNS penetrant inverse agonist **37** (AZ-GHS-22) (IC_50_ (affinity) = 6.7 nM) (Figure 11A), bearing a morpholine moiety in position 5 of the phenyl ring, were identified. Interestingly, compound **36**, but not **37**, reduced acute food intake in wild-type mice. This effect was not observed in GHS-R1a knockout mice, demonstrating the involvement of such a receptor in the mechanism of action.

New potent GHS-R1a inverse agonists bearing the 2-aminoalkyl nicotinamide scaffold were identified by Asubio Pharma.^76^ Optimization of the 2-aminoalkyl and 5-(*N*-propyl)pyrazolyl groups of the hit compound **38** (IC_50_ (affinity) = 84 nM) afforded the lead **39** (IC_50_ (affinity) = 0.96 nM) (Figure 11B), characterized by an azabicyclo ring at the 5-position and by a (2,3-(dihydrobenzofuran)methylamine at the 2-position of the pyridine ring. It peripherally blocked ghrelin-induced food intake and showed anorexigenic effects in mice.

The low oral bioavailability of **39** prompted the optimization of its structure through the modification of the substituents in positions 2 and 3 of the pyridine ring to improve the metabolic stability and in position 5 to reduce the molecular weight. The peripherally acting compound **40** (IC_50_ (affinity) = 6.6 nM) and the brain-penetrant derivative **41** (IC_50_ (affinity) = 0.28 nM) (Figure 11B), both endowed with oral bioavailability higher than **39**, were evaluated in rat models of obesity.^77^ Compound **41** showed higher efficacy than **40** in abolishing weight gain, indicating that the antiobesity effects of these inverse agonists might be attributed to the suppression of CNS GHS-R1a activity.

Pfizer identified the HTS-hit **42** (*K*_i_ = 213 nM) bearing a spiro-azetidine-piperidine scaffold,^78^ which was optimized to the centrally acting GHS-R1a inverse agonist lead **43** (*K*_i_ = 6.3 nM) (Figure 11C).^79^ This last compound induced insulin secretion in a glucose-dependent manner in islet cells.^80^ However, its poor selectivity over other targets, such as α_2a_ and α_2c_ adrenergic, D_2_-like dopaminergic and H_1_ histaminergic receptors, as well as hERG channels, and inadequate physicochemical properties and safety profiles prevented its further development. A physicochemistry-based strategy to improve the PK properties and to reduce both the off-target activity and CNS penetration of the compounds, with the aim to limit the CNS-based side effects, led to the identification of **4** (PF-5190457) (*K*_i_ = 4.4 nM) (Figure 6), characterized by an imidazothiazole group and an *R* configurated 6-methyl-4-pyrimidinyl indane linked to the spiro-azetidine-piperidine scaffold. Compound **4** behaved as a potent and very selective peripherally acting GHS-R1a inverse agonist, with an improved safety profile and PK properties. For its pharmacological profile, **4** progressed to human clinical trials.^80^ Recently, its main circulating hydroxy metabolite **44** (PF-6870961) (Figure 11C) has been identified by LC-MS/MS in human plasma.^81^ Considering the promising result obtained from clinical studies with **4** and its therapeutic potential in the alcohol abuse treatment (see section 4.7), a synthetic chemistry route was developed to obtain a sufficiently large amount of **44**, in order to evaluate the properties and pharmacological profile of this metabolite.^82^

Starting from lead **43**, another series of spiro-azetidine-piperidine derivatives was also developed to improve potency, PK, and the safety profile by emphasizing increased polarity of the compounds.^83^ Compound **45** (*K*_i_ = 9.2 nM) (Figure 11C), endowed with an optimal combination of potency, polarity, and *in vivo* PK properties, was obtained. However, owing to pH-dependent chemical instability of the ortho-carboxamide function, its further development was discontinued.

**Figure 12:**
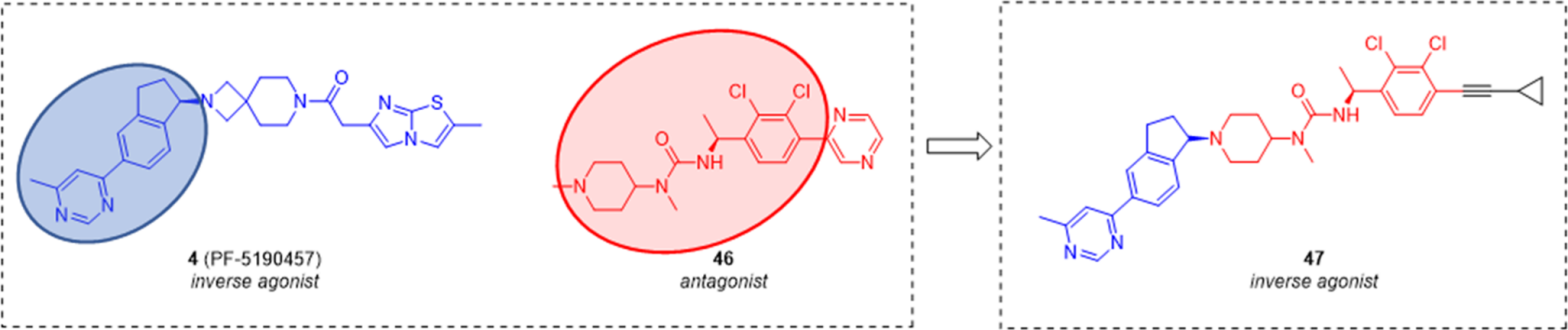
Chemical structure of GHS-R1a inverse agonist **47**, in which the structure of the inverse agonist **4** was combined with that of the competitive antagonist **46**.

More recently, the structure of the peripherally active inverse agonist **4** has been combined with that of the substituted asymmetric urea compound **46** (Figure 12), behaving as a potent competitive GHS-R1a antagonist with a favorable PK profile,^84^ by a chimeric drug design approach,^85^ generating an “imidothiazol”, “piperidine”, and “spiro-piperidine” structure series. From SAR and structure–property relationship studies, compound **47** (Figure 12) was identified as a potent GHS-R1a antagonist (IC_50_ = 68 nM) and inverse agonist (EC_50_ = 29 nM) in cellular assays.^85^ It also showed high CNS penetration and moderate oral bioavailability in rat. In *in vivo* studies it effectively reduced food intake in mice. Further studies are needed to better evaluate the potential of such a compound as a therapeutic agent for the treatment of metabolic disorders associated with obesity.

**Figure 13:**
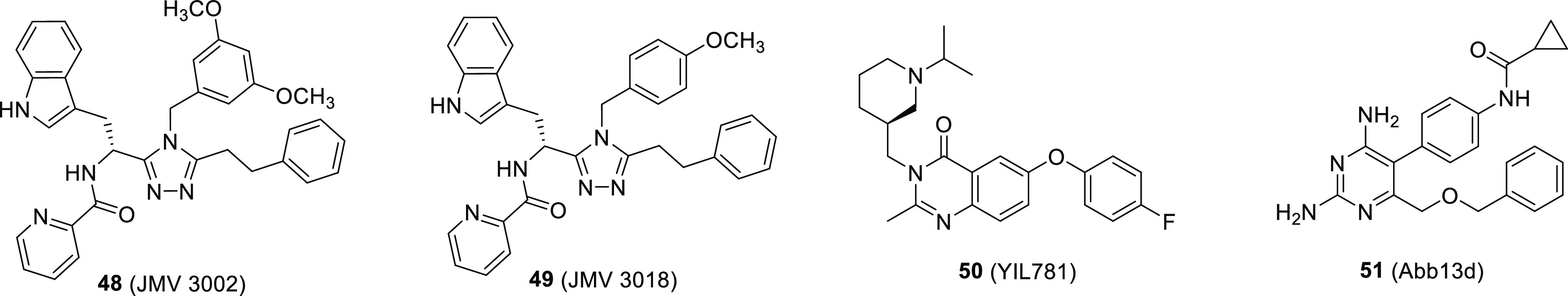
Chemical structure of GHS-R1a biased ligands **48**–**51**.

A recent successful approach concerns the analysis of the properties of small molecules, originally reported as GHS-R1a inverse agonists or antagonists, in different signaling pathways, to evaluate whether they show functional selectivity. For this purpose, the pharmacological behavior of several GHS-R1a synthetic ligands was revisited by evaluating their selectivity toward several G-protein isoforms and G-protein-independent pathways. Some of them, such as the above-discussed compound **29** (Figure 10), as well as JMV 3002 (**48**), and JMV 3018 (**49**) (Figure 13), behaved as biased agonists for Gq activation and IP production and antagonists for β-arrestin recruitment, ERK1/2 phosphorylation, and Gi2, Gob activation. Instead, compound **32** (Figure 10) proved to be an inverse agonist only toward Gq activation and IP production and was silent toward G13 activation.^8^

In a more recent study, compound **29** proved to decrease the constitutive activity of GHS-R1a by specifically reducing the GHS-R1a basal internalization, without affecting ERK1/2 basal phosphorylation state and β-arrestin recruitment, suggesting that it might represent a specific biased inverse agonist.^61^

Such an approach also highlighted that compound **50** (YIL781) (Figure 13), previously described by Bayer as a GHS-R1a antagonist,^86^ behaved as a biased ligand, selectively activating Gαq/11 and Gα12, and devoid of intrinsic activity for β-arrestin recruitment and other G-proteins activation.^41^ In *in vivo* studies, it demonstrated to decrease gastric emptying and to increase food intake. In contrast, the Abbott antagonist **51** (Abb13d)^87^ (Figure 13) proved to be a Gαq/11 inverse agonist, decreasing both these *in vivo* effects. This result suggests that Gαq/11 activation promotes homeostatic food intake, while reduction of gastric emptying is induced by neutral antagonism or inverse agonism at the other pathways.^41^

### 3.3 GHS-R1a Ligands for Molecular Imaging

Recent efforts have been devoted to the development of PET imaging agents targeting GHS-R1a, with the aim to image and target this receptor for diagnosis and treatment of different diseases, especially cancer and cardiovascular disorders, as well as for the study of the localization and functions of GHS-R1a in the body. Though several studies have been focused on ghrelin analogues and peptide derivatives,^88−90^ as stated above, in this section we will only discuss radiopharmaceutical nonpeptide small molecules. In particular, fluorine-containing molecules with high GHS-R1a affinity have been identified to be radiolabeled with ^18^F, one of the most common radioisotopes used for PET imaging.^91^

**Figure 14:**
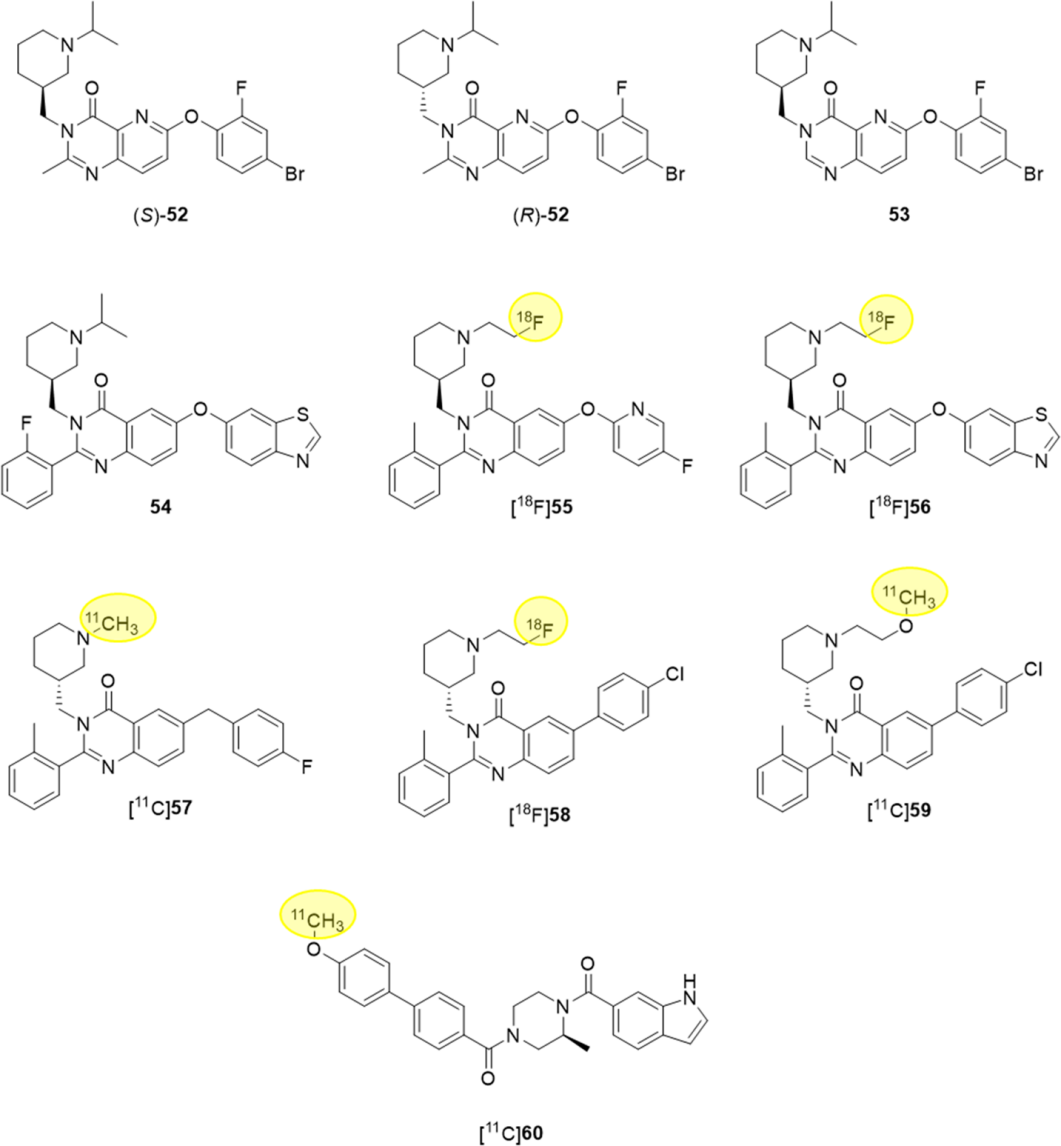
Chemical structure of GHS-R1a ligands **52**–**60**, potentially useful for molecular imaging.

Within a series of derivatives bearing an azaquinazolinone nucleus, one of the scaffolds used in the design of potent GHS-R1a ligands,^92^ the fluorinated derivatives (*S*)-**52** (IC_50_ (affinity) = 2.2 nM), (*R*)-**52** (IC_50_ (affinity) = 3.9 nM), and **53** (IC_50_ (affinity) = 2.7 nM) (Figure 14), endowed with good bioavailability and able to cross the blood-brain barrier (BBB), have recently been identified as suitable compounds for ^18^F-labeled PET radiotracers for brain imaging.^93^

A parent class of small molecules targeting GHS-R1a is represented by quinazolinones,^86^ for which an extensive SAR study has recently been carried out to develop derivatives with very high affinity for GHS-R1a and moderate cLogD. Among them, the fluorinated compound **54** (Figure 14) emerged as the ligand endowed with the highest GHS-R1a binding affinity reported until then (*K*_i_ = 20 pM), but unfortunately, attempts to radiolabel this derivative were unsuccessful. However, the lead compounds **55** and **56** (Figure 14), showing nanomolar affinity (IC_50_ (affinity) = 20.6 and 9.3 nM, respectively), were successfully ^18^F-radiolabeled and might represent potential tools for cancer diagnosis and therapy.^94^

Other nonpeptide PET tracers for GHS-R1a are represented by [^11^C]**57** (*K*_i_ = 22 nM) (Figure 14), showing moderately specific binding to GHS-R1a in *in vivo* mouse brain but not in periphery,^95^ and the more recently radiosynthesized [^18^F]**58** (*K*_i_ = 16 nM), [^11^C]**59** (*K*_i_ = 4 nM), and [^11^C]**60** (*K*_i_ = 7 nM) (Figure 14).^96^ Among these, [^11^C]**60** might be considered a useful PET tracer for *in vivo* imaging of GHS-R1a in pancreas, showing specific binding to GHS-R1a in mice pancreas and good uptake.

## 4 Pharmacological Potential of GHS-R1a Ligands

Due to the wide distribution of GHS-R1a in CNS and in periphery, and its involvement in several physiological functions, ligands modulating GHS-R1a signaling pathways might be beneficial to the treatment of numerous disorders, including anorexia, cachexia, sarcopenia, gastrointestinal and metabolic diseases, neurological and neurodegenerative disorders, pain, and substance use disorders (Table 1).^12,53^ The effects of small molecules behaving as GHS-R1a agonists, antagonists, and inverse agonists on such pathologies will be discussed in this section. Moreover, molecules potentially useful as diagnostic compounds, such as the orally active GHS-R1a agonist **26**, recently commercialized as Macrilen for the diagnosis of GH deficiency in adults, being reliable, safe, well tolerated, and able to potently and selectively stimulate the GH release, deserve to be mentioned.^97−101^

**Table 1:** GHS-R1a Nonpeptide Ligands Showing Therapeutic Potential in Preclinical and/or Clinical Studies

compound	GHS-R1a functional behavior	potential therapeutic applications
**3** (ibutamoren, MK-0677)	agonist	sarcopenia,^120,121^
		Alzheimer’s disease^170^
**4** (PF-5190457)	inverse agonist	metabolic diseases,^155^
		alcohol use disorders^192−194^
**5** (capromorelin, CP-424391)	agonist	gastrointestinal diseases^132−135^
**6** (anamorelin, ONO-7643)	agonist	cancer cachexia, anorexia^106,107,109,111−117^
**7** (ulimorelin, TZP-101)	agonist	gastrointestinal diseases^136−141^
**11**	agonist	cachexia^59^
**14**	agonist	cachexia^60^
**18**	biased ligand	cachexia^56^
	(G-protein agonist)	
**26** (macimorelin, JMV 1843)	agonist	diagnosis of GH deficiency,^97−101^
		epilepsy^165,167^
**29** (JMV 2959)	antagonist or biased ligand	obesity,^149,150^
	(Gq agonist, β-arrestin, ERK1/2 phosphorylation and Gi2, Gob antagonist)	substance use disorders^180−189^
**33**	inverse agonist	obesity^72^
**36** (AZ-GHS-38)	inverse agonist	obesity^75^
**41**	inverse agonist	obesity^77^
**47**	inverse agonist	obesity^85^
**48** (JMV 3002)	antagonist or biased ligand	obesity^149^
	(Gq agonist, β-arrestin, ERK1/2 phosphorylation and Gi2, Gob antagonist)	
**50** (YIL-781)	biased ligand	metabolic diseases,^86,148^
	(Gαq/11 and Gα12 agonist, β-arrestin antagonist)	substance use disorders^190,191^
**51** (Abb13d)	Gαq/11 inverse agonist	gastrointestinal diseases^41^
**61** (HM01)	agonist	cancer cachexia,^103,104,109^
		gastrointestinal diseases,^129,130^ neurotoxicity,^164^ Prader–Willi syndrome,^161^
		Parkinson’s disease,^174,175^ pain^179^
**62** (Z-505)	agonist	cancer cachexia, anorexia^107,108,110^
**63** (HM02)	agonist	gastrointestinal diseases^129^
**65** (LY444711)	agonist	Alzheimer’s disease^169^

### 4.1 Anorexia and Cachexia

**Figure 15:**
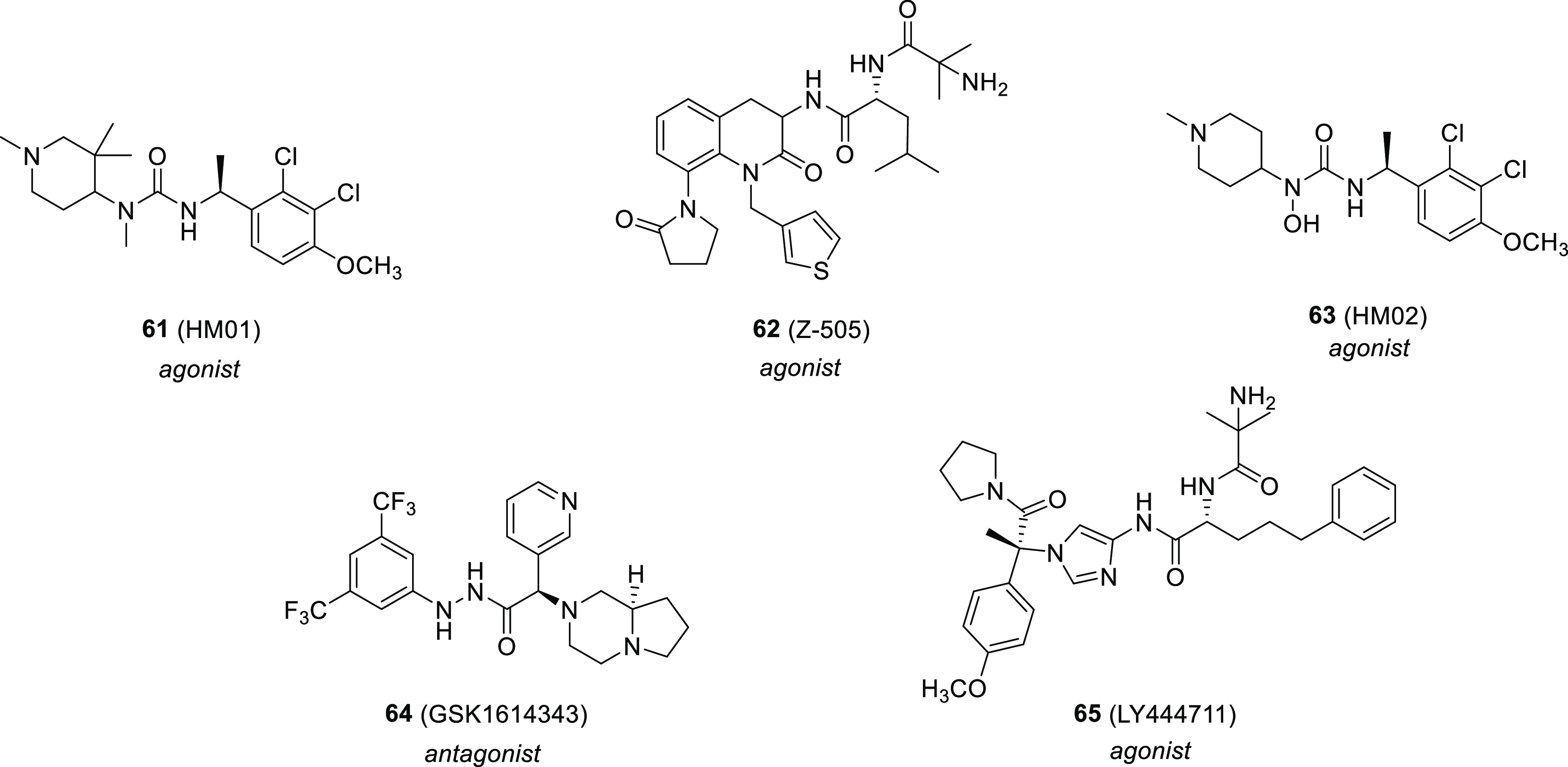
Chemical structure of GHS-R1a ligands **61**–**66**.

Due to the established lipogenic and orexigenic effects of AG, various preclinical and clinical studies were performed and supported the beneficial role of AG or GHS-R1a agonists in the treatment of anorexia and cachexia.^12,102^ Prevention of tissue wasting and increased food intake have been observed in a series of studies evaluating the role of known GHS-R1a agonists, such as compound **6**, HM01 (**61**), and Z-505 (**62**) (Figure 15) in rodents bearing tumors associated with cachexia.^103−108^ Recently, it has been reported that both compounds **6** and **61** potently induce Ca^2+^ mobilization, but as compound **6** is more effective in the β-arrestin recruitment and GHS-R1a internalization, it is potentially more susceptible than compound **61** to treatment-induced tolerance, highlighting the importance of signaling bias characterization in the future development of GHS-R1a ligands.^109^ Compound **62** was also demonstrated to decrease anorexia after total gastrectomy in rats.^110^

Several clinical studies have reported that GHS-R1a agonists can be effective in improving anorexia and cachexia with limited side effects in healthy young adults and cancer patients, and in particular compound **6** represents a promising agent for the treatment of such pathologies.^111−115^ In December 2020, it was approved in Japan for cancer cachexia.^116^ Moreover, a very recent trial has reported its efficacy in association with nutrition counselling and physical activity in improving cancer-related fatigue, one of the most common symptoms in advanced cancer patients.^117^

### 4.2 Sarcopenia

Due to the low ghrelin levels found in elderly subjects with sarcopenia,^118^ GHS-R1a agonists might be beneficial in the treatment of this disease. The Japanese herbal medicine rikkunshito, acting as a ghrelin-potentiator, was able to inhibit age-related sarcopenia in a mouse model of senescence.^119^ Oral administration of the agonist **3** for 12 months in a randomized double-blind placebo-controlled clinical trial prevented lean mass loss and caused an increase of IGF-1 and GH levels in healthy elderly humans with respect to younger adults with few adverse effects.^120^ Serum IGF-1 levels were also increased in hemodialysis individuals, suggesting the beneficious potential of compound **3** for end-stage renal disease and chronic kidney disease patients with protein-energy wasting.^121^

### 4.3 Gastrointestinal Diseases

One of the first functions identified in the study of ghrelin signaling is the effect on the gastrointestinal tract, where AG stimulates gastric motility and acid secretion in rats.^122^ Treatment with the ghrelin-potentiator rikkunshito was also demonstrated to ameliorate symptoms of dyspepsia.^123^ One of the most clinically studied GHS-R1a agonists for gastric motility diseases and constipation is the pentapeptide relamorelin (RM-131, BIM-28163).^124−127^ However, focusing our attention on nonpeptide small molecule, the centrally acting GHS-R1a agonist **61** proved to potently induce colorectal motility and bowel emptying, through the stimulation of the lumbosacral spinal defecation center.^128^ This compound, and its more peripherally acting analogue HM02 (**63**) (Figure 15), contrasted the delayed gastrointestinal transit induced by abdominal surgery in a rat model of postoperative ileus, whereas in a rodent defecation assay only ligand **61** was able to significantly increase the weight of fecal pellets. These results suggest that a peripheral site of action is involved in the stimulation of gastrointestinal transit induced by synthetic GHS-R1a agonists, while the increase of the weight of fecal pellets is mediated by a centrally located site.^129^ Compound **61** also promoted motion-induced emesis more effectively than compound **63** in *suncus murinus*, suggesting that this effect is centrally induced, probably by the activation of GHS-R1a of the paraventricular hypothalamic nucleus.^130^

Compound **5**, another brain penetrant GHS-R1a agonist recently approved for veterinary use in cats and dogs,^131^ effectively accelerated gastric emptying in mice^132^ and stimulated defecation in a rat model of low fiber-induced constipation.^133^ This compound also induced colon contractions and spontaneous defecation in spinal cord-injured rats.^134^ A phase 1 clinical trial demonstrated the safety profile and tolerability of compound **5** in constipated spinal cord-injured patients.^135^

Gastrointestinal motility was also accelerated by the synthetic macrocyclic agonist **7** both in preclinical and clinical studies.^136−138^ However, this compound failed to meet end points in two multicenter placebo-controlled phase 3 trials in postoperative ileus.^139^ Recently, its effects on stomach and colon motility of healthy volunteers have been investigated and the results suggested that the stomach is the main site of AG action in humans, as **7** is a potent gastric prokinetic devoid of activity in the colon.^140^ Compound **7** also proved to be safe and effective in the treatment of enteral feeding intolerance.^141^

### 4.4 Metabolic Diseases

Considering the well-known role of ghrelin in inducing adiposity and stimulating appetite^142,143^ as well as in the regulation of glucose metabolism,^29,144^ different active vaccines based on the ghrelin structure have been developed over the years to prevent obesity.^145−147^ GHS-R1a antagonists or inverse agonists might also represent promising agents for the management of metabolic diseases. Over the years, GHS-R1a antagonists with different molecular scaffolds proved to be potentially beneficial for disorders such as obesity, diabetes, and hyperglicemia.^53^ In particular, quinazolinone derivatives, including ligand **50**, were reported to induce weight loss in diet-induced obese mice. This compound also improved glucose tolerance associated with obesity by increasing insulin release.^86,148^ However, more recently, it has demonstrated to decrease gastric emptying and increase food intake in mice. As discussed in section 3.2, such an effect might be due to its biased behavior.^41^

Different 1,2,4-triazole antagonists, including the aforementioned **29**, **33**, and **48**, were able to inhibit food intake in rodents.^66,149,150^ In contrast, the carbohydrazide antagonist GSK1614343 (**64**) (Figure 15)^151^ surprisingly enhanced food intake and weight gain in dogs and rats,^152^ indicating that the benefit of antagonists in the metabolic disorders needs to be further investigated. A more promising strategy to contrast these pathologies is represented by inverse agonists, owing to their ability to reduce the constitutive GHS-R1a activity.^74,153^

Among the aforementioned acylureas developed by AstraZeneca, the CNS penetrant inverse agonist **36** but not the non-CNS penetrant **37** reduced acute food intake in wild-type mice.^75^ Accordingly, the nicotinamide brain-penetrant compound **41** showed higher efficacy than the peripherally acting derivative **40** in reducing weight gain (see section 3.2), indicating that the antiobesity effects of these inverse agonists might be attributed to the suppression of CNS GHS-R1a activity.^77^ Two more recently reported inverse agonists (structures not disclosed) demonstrated to decrease food intake in mice. One of them also caused hypoglycemia and reduced body weight and triglyceride levels.^154^

Among the spiro-azetidine-piperidines, the already mentioned orally bioavailable GHS-R1a inverse agonist **4**(80) reached the clinical trials, being able to increase insulin secretion both in the human pancreas and Langerhans islets. In healthy people, it reduced stomach motility and evacuation, as well as GH secretion, and induced hypoglycemia.^155^

A metabolic disorder caused by genetic defects is represented by Prader–Willi syndrome (PWS), which is characterized by several symptoms, including obesity, hyperphagia, low GH, neonatal hypoglycemia, infertility, and accelerated mortality.^156,157^ Though many studies suggest that high ghrelin levels might be responsible for hyperphagia and obesity in patients with PWS,^158^ this association has never been demonstrated. On the contrary, other known effects of ghrelin, such as hyperglycemia and increase of GH secretion, muscle mass and strength, and survival,^1,159^ as well as its anxiolytic and antidepressant actions^34,160^ might be beneficial for PWS. Interestingly, the GHS-R1a agonist **61**, daily administered for 2 weeks, markedly enhanced survival of Snord116del neonatal mice, a preclinical model of PWS. These results prompt to explore in depth the therapeutic potential of GHS-R1a agonists in limiting mortality in PWS, especially before the hyperphagic nutritional phase starts.^161^

### 4.5 Neurological and Neurodegenerative Disorders

As mentioned above, AG signaling plays a crucial role in the CNS functions, such as synaptic plasticity, learning, memory, and neurogenesis,^32,162,163^ supporting the potential use of GHS-R1a agonists in the treatment of neurological and neurodegenerative disorders.^26^ The neuroprotective effects of GHS-R1a agonists were also observed in cancer patients treated with neurotoxic chemotherapy. Indeed, the brain penetrant compound **61** was able to attenuate cisplatin-, oxaliplatin-, and bortezomib-induced neurotoxicity in mice.^164^

#### 4.5.1 Epilepsy

Recently, ghrelin and GHS-R1a agonists are gaining substantial recognition as an innovative approach to treat epilepsy.^37^ The full agonist **26** proved to decrease the seizure severity score both in acutely 6 Hz corneal electrical stimulated mice and in fully kindled mice but not in GHS-R1a knockout mice. This effects were not observed after administration of the antagonist **29**.^165^ On the contrary, kindled mice treated with the aforementioned biased ligand **50**, selectively activating Gαq/11 and Gα12 and being devoid of intrinsic activity for β-arrestin recruitment, showed more severe and longer seizures, suggesting that the anticonvulsive effect of ligand **26** might be due to the activation of the β-arrestin signaling pathway.^166^ Very recently, compound **26** has proved to induce anticonvulsant effects in drug-refractory intrahippocampal kainic acid mouse model of epilepsy, suggesting its potential use in pharmacoresistant epilepsy.^167^

#### 4.5.2 Alzheimer’s Disease

Several studies have reported the effects of GHS-R1a agonists on Alzheimer’s disease (AD) symptoms.^168^ Improved cognitive functions and reduced cerebral inflammation and beta-amyloid levels have been induced by the oral administration of the GHS-R1a agonist LY444711 (**65**) (Figure 15) in a mouse model of AD.^169^

More recently, the agonist **3** has been reported to reduce Aβ deposition, neurodegeneration, and neuroinflammation in a mouse model of early stage of AD.^170^ However, this compound failed to prevent hippocampal lesions in a mouse AD model and to mitigate cognitive impairment in a clinical trial with AD patients, suggesting its ineffectiveness alone for the treatment of AD.^171,172^

#### 4.5.3 Parkinson’s Disease

The observation that ghrelin could prevent the degeneration of striatal dopaminergic neurons, expressing GHS-R1a, induced by the neurotoxin 1-methyl-4-phenyl-1,2,3,6-tetrahydropyridine,^173^ supports the potential of GHS-R1a agonists in the management of Parkinson’s disease (PD). In a 6-hydroxydopamine rodent model of PD, the brain penetrant agonist **61** was able to normalize the decreased 4 h fecal output and the gastric emptying blocked by levodopa.^174^ Following chronic administration, the same compound ameliorated several nonmotor symptoms of PD including body weight loss, fecal weight and water content, water consumption, as well as enhanced food intake. These findings suggest a potential benefit of GHS-R1a agonists to alleviate nonmotor symptoms in PD patients with gastrointestinal disorders.^175^

### 4.6 Pain

Due to its anti-inflammatory effects, ghrelin has been demonstrated to show antinociceptive activity in models of inflammatory and neuropathic pain.^176,177^ Interestingly, it has been reported that these effects can also be mediated by different central pathways.^178^

Recently, the GHS-R1a agonist **61** has shown analgesic effects in a rat model of noninflammatory visceral hypersensitivity and somatic mechanical allodynia, suggesting the activation of GHS-R1a signaling as a potential novel approach for the treatment of visceral and somatic pain.^179^

### 4.7 Substance Use Disorders

GHS-R1a blockade has been suggested as a promising approach for the treatment of substance use disorders.^33,36^ The GHS-R1a antagonist **29** demonstrated to decrease alcohol-, morphine-, nicotine-, cocaine-, amphetamine-, methamphetamine-, fentanyl-, or cannabinoid-induced conditioned place preference and/or locomotor stimulation,^180−188^ as well as to reduce alcohol-, amphetamine-, morphine-, nicotine-, or cocaine-induced dopamine release in the nucleus accumbens and/or the ventral tegmental area in rodents.^186−189^

Moreover, the GHS-R1a biased ligand **50** significantly reduced hyperlocomotion in a dopamine-transporter knockout mouse model,^190^ as well as in cocaine-sensitized mice, suggesting that the blockade of β-arrestin recruitment might be required for this effect.^191^

Interesting results have recently been obtained with the inverse agonist **4**, which reached clinical trials for its potential in the treatment of alcohol use disorders. Safety and tolerability of this compound, coadministered with alcohol in active heavy alcohol drinking patients, were demonstrated in preclinical safety experiments and phase 1b clinical studies. Compound **4** was also suggested to decrease alcohol cue-induced craving, which represents a risk factor for relapse in subjects with alcohol use disorders.^192−194^

## 5 Conclusions and Prospects

The considerable attention of researchers from both pharmaceutical companies and academies concerning the modulation of the ghrelin system by using GHS-R1a ligands is demonstrated by the large number of papers published in the last years. This interest is due to the fact that GHS-R1a represents a promising target for the treatment of numerous disorders. In particular, while agonists have shown efficacy in the management of anorexia, cachexia, sarcopenia and gastrointestinal diseases, epilepsy, and pain and neurodegenerative disorders, antagonists and inverse agonists have proved to have potential in the treatment of substance use disorders and metabolic diseases, including obesity and diabetes. Over the years, compounds with different molecular scaffolds have been identified, and some of them have been extensively studied in clinical trials. In this regard, inverse agonists have demonstrated to be more effective candidates than antagonists for preclinical and clinical studies, as they are able to reduce the unusually high constitutive activity of GHS-R1a.

Another important aspect concerns the development of PET imaging GHS-R1a radiolabeled ligands, potentially useful for diagnosis and treatment of cancer and cardiovascular diseases as well as for the study of GHS-R1a localization and functions in the body.

The recently resolved structures of GHS-R1a bound to ghrelin or potent ligands have greatly improved the knowledge of the molecular mechanism for GHS-R1a recognition and activation and provided useful information for the design of new GHS-R1a selective drugs.

A further strategy for the discovery of new drugs has originated from the assessment of the functional profile of small molecules in different signaling pathways of GHS-R1a to evaluate whether they behave as biased ligands. This approach has helped to improve the knowledge of the biological functions associated with each pathway and to identify functionally selective compounds, which might be useful for the treatment of diseases associated with the modulation of a specific signaling pathway, avoiding potential side effects.

Overall, this perspective aims to provide information which might help to develop new potent GHS-R1a agonists, antagonists, and inverse agonists to clarify the role played by GHS-R1a in the diseases in which it is involved and to identify new pharmacological tools potentially useful for their treatment.

## Abbreviations Used

AD: Alzheimer’s Disease
AG: active acyl-ghrelin
BBB: blood-brain barrier
CNS: central nervous system
DAG: desacyl-ghrelin
GH: growth hormone
GHSs: growth hormone secretagogues
GOAT: ghrelin *O*-acyltranferase
GPCR: G protein-coupled receptor
HTS: high-throughput screening
MRAP2: melanocortin receptor accessory protein 2
LEAP2: liver-expressed antimicrobial peptide 2
IGF-1: insulin-like growth factor-1
IP: inositol 1-phosphate
NMR: nuclear magnetic resonance
PD: Parkinson’s disease
PET: positron emission tomography
Pgp: P-glycoprotein
PK: pharmacokinetic
PWS: Prader–Willi syndrome
SAR: structure–activity relationship
